# Non‐invasive quantitative assessment of induced component displacement can safely and accurately diagnose tibial component loosening in patients: A prospective diagnostic study

**DOI:** 10.1002/ksa.12299

**Published:** 2024-05-31

**Authors:** George S. Buijs, Arthur J. Kievit, Maaike A. Ter Wee, Caroline Magg, Johannes G. G. Dobbe, Geert J. Streekstra, Matthias U. Schafroth, Leendert Blankevoort

**Affiliations:** ^1^ Department of Orthopedic Surgery and Sport Medicine Amsterdam UMC, location AMC Amsterdam The Netherlands; ^2^ Amsterdam Movement Sciences Musculoskeletal Health Meibergdreef 9 Amsterdam The Netherlands; ^3^ Department of Biomedical Engineering and Physics Amsterdam UMC, location AMC, University of Amsterdam Amsterdam The Netherlands; ^4^ Quantitative Healthcare Analysis (QurAI) Group, Informatics Institute University of Amsterdam Amsterdam The Netherlands

**Keywords:** aseptic loosening, computed tomography, diagnostic test, imaging, knee arthroplasty

## Abstract

**Purpose:**

Aseptic loosening often requires major, expensive and invasive revision surgery. Current diagnostic modalities merely show indirect signs of loosening. A recent proof of concept study proposed a non‐invasive technique for the quantitative and visual assessment of implant movement as a diagnostic aid for tibial component loosening. The primary research question addressed is whether this novel diagnostic modality can safely and effectively aid the diagnosis of aseptic loosening.

**Methods:**

This clinical study included patients suspected of aseptic total knee arthroplasty (TKA) loosening listed for revision surgery and asymptomatic patients. Safety was evaluated using a numerical rating scale (NRS) for discomfort and by registration of adverse events. Feasibility was assessed by recording the duration and ease of the procedure. Intra‐ and interrater reliability were evaluated. In symptomatic patients, diagnostic accuracy metrics were evaluated with intra‐operative assessment as a reference test.

**Results:**

In total, 34 symptomatic and 38 asymptomatic knees with a TKA were analysed. The median NRS for discomfort during loading was 6 (interquartile range [IQR]: 3.75–7.00) in symptomatic patients and 2 (IQR: 1.00–3.00) in asymptomatic patients. No adverse events were reported. The majority of users found the use of the loading device easy. The median time spent in the computed tomography room was 9 min (IQR: 8.00–11.00). Excellent to good intra‐ and interrater reliabilities were achieved. Diagnostic accuracy analysis resulted in a sensitivity of 0.91 (95% confidence interval [CI]: 0.72–0.97) and a specificity of 0.72 (95% CI: 0.43–0.90).

**Conclusions:**

The proposed diagnostic method is safe, feasible, reliable and accurate in aiding the diagnosis of aseptic tibial component loosening.

**Level of Evidence:**

Level II.

**Abbreviations**
AUC: area under the curve
CT: computed tomography
ICC: interclass correlation coefficient
IQR: interquartile range
KOOS: Knee Injury and Osteoarthritis Outcome Score
NPV: negative predictive value
NRS: numerical rating scale
PET‐CT: positron emission tomography combined with CT
PPV: positive predictive value
ROC: receiver operating characteristic
rTKA: revision total knee arthroplasty
SD: standard deviation
SPECT/CT: single‐photon emission computed tomography combined with CT
TKA: total knee arthroplasty
VIF: variance inflation factor

## INTRODUCTION

Following infection, aseptic loosening is reported to be the second most common cause of Total Knee Arthroplasty (TKA) failure [10, 17]. Aseptic loosening often requires major, expensive and invasive revision TKA (rTKA) surgery. The diagnosis of aseptic loosening is challenging as there is no consensus in the available literature regarding a standardized diagnostic work‐up including a specific diagnostic test [4, 7].

If a patient presents at the outpatient clinic with symptoms indicative of TKA loosening, for instance, pain during weight‐bearing activities, conventional radiography is typically the first imaging modality used to assess bone resorption and TKA component migration as signs of loosening [3, 6]. The sensitivity and specificity of plain radiography are inadequate, particularly in cases of early and subtle but clinically relevant loosening [12].

Although additional imaging modalities like computed tomography (CT), 3‐phase bone scintigraphy, positron emission tomography combined with CT (PET‐CT) and single‐photon emission computed tomography combined with CT (SPECT/CT) are used, the American College of Radiology does not recommend them for evaluating pain post knee arthroplasty once the infection is ruled out [23]. Nuclear imaging modalities measure secondary and aspecific effects, such as increased bone turnover and osteoclastic activity, while exposing patients to high radiation dosages (4–7 mSv) [2, 4, 12, 18]. Accurate diagnosis is crucial to prevent unwarranted revision surgeries in patients misdiagnosed with loosening of the TKA, while also ensuring that patients with unrecognized loosening are not mistakenly excluded from necessary revision procedures. Additionally, it prevents patients from enduring needless delays in receiving appropriate care with a correct diagnosis [20, 23].

As a solution to improve the diagnostic work‐up, Kievit et al. proposed and evaluated a less cumbersome alternative [14]. This method employs a loading device to exert up to 20 N m on the knee in alternating valgus and varus directions. In each direction, a CT scan is made (exposing patients to an estimate of 1.2 mSv). Advanced imaging analysis then quantifies and visualizes the tibial component's displacement between the two directions relative to the bone. This pilot study demonstrated reliable and reproducible results in a cadaveric setting [14]. A patient study was deemed necessary to further evaluate the diagnostic potential and clinical usability of this method.

Our primary research question was: What are the diagnostic accuracy metrics of the proposed modality? Our secondary research questions were: (1) Is the method safe for application in a clinical setting? (2) Is the method feasible for clinical use, in terms of duration of evaluations, ease and successfulness of use? (3) What is the intra‐ and interrater reliability of this method?

For evaluating the diagnostic accuracy metrics, the subsequent questions were posed: (1) What extent of induced tibial component displacement is observed in both symptomatic and asymptomatic TKA patients? (2) What was the most optimal threshold for induced displacement to classify a tibial component as either fixed or loose, with the intraoperative observation as reference test and what are the consequent diagnostic accuracy metrics? And, additionally, to what extent are Patient Reported Outcome Measures (PROMs) correlated to the induced displacement of the tibial component?

This follow‐up study hypothesizes that the proposed method is safe, feasible, reliable and accurate to aid the diagnosis of loosening of the tibial component.

## METHODS

### Ethical statement

This study was reported in accordance with the Code of Ethics of the World Medical Association (Declaration of Helsinki) for experiments involving humans, and the Recommendations for the Conduct, Reporting, Editing and Publication of Scholarly Work in Medical Journals.

### Patient screening and inclusion

In this prospective diagnostic test accuracy study, conducted by the Amsterdam UMC, seven affiliated hospitals were invited to refer patients scheduled for rTKA for the diagnosis of aseptic TKA loosening for inclusion in the study. The diagnosis of aseptic loosening was established per local protocol, including physical examination and the exclusion of infection by C‐reactive protein and joint aspiration if needed [13]. Patients referred solely for the definitive diagnosis of aseptic loosening were included in the symptomatic group.

Local registries of TKA patients were screened for eligible patients for the asymptomatic group. Patients who reported complete satisfaction with their existing TKA and had no complaints during their most recent outpatient visit to the Amsterdam UMC were considered asymptomatic. These individuals were subsequently contacted and invited to participate in the study. Those who consented were then included in the asymptomatic group.

### Safety

To assess the safety of this method, patients were asked to rate the discomfort they experienced during and after loading of the knee on a numeric rating scale (NRS) of 0–10, with 0 indicating no pain or discomfort and 10 indicating extreme pain or discomfort. Additionally, adverse events were recorded.

### Feasibility

To evaluate the feasibility, medical staff applying and operating the loading device were asked to rate the ease of positioning of the loading device, attachment of straps and application of moment as either easy, intermediate or difficult. Additionally, the actual applied moment in both valgus and varus directions and the total time spent by the patient in the CT room were registered.

### Reliability

To evaluate the reliability of the measurements resulting from the 3D analysis software, an intra‐ and interrater reliability assessment was conducted. Three distinct raters, all trained but two experienced (G.B. and A.W.) and one inexperienced (C.M.), analysed all the scans separately. One rater (G.B.) analysed a random sample of 10 scans two times, with a 30‐day time interval between the analyses. All raters were blinded for the results generated by the other readers, as well as for the outcome of the reference test employed for the diagnostic accuracy analysis.

### Diagnostic accuracy

#### Index test: Measurement of induced displacement

##### Hardware: Loading device

**Figure 1:**
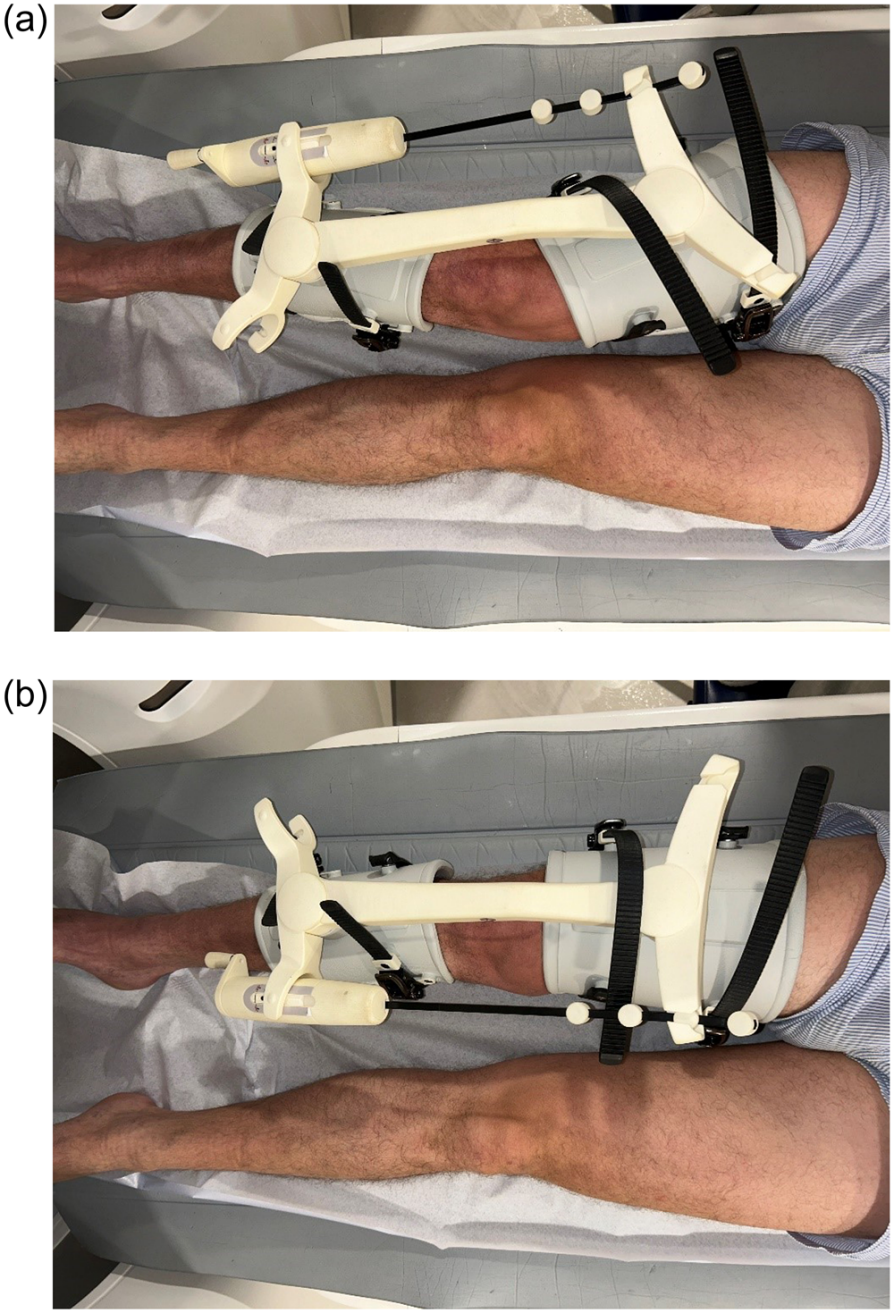
(a, b) Picture of the knee in CT scanner with the loading device in valgus and varus. CT, computed tomography.

A patented loading device was used to apply a valgus‐ and varus moment on the knee whilst conducting a CT scan in both situations (Figure 1) [5, 21]. This device is designed to apply a bending moment on the knee in the frontal plane of the tibia of 20 Nm with the knee in 20‐degree flexion using the principle of four‐point bending. The 20‐degree flexion is to relax the posterior capsule and cruciate ligaments. The externally applied bending moment is internally balanced by the combination of (1) a compressive force in either the medial compartment (varus moment) or lateral compartment (valgus moment) and (2) the forces in the lateral collateral ligament and capsule or in the medial collateral ligament and capsule, respectively.

The compressive forces on the medial and lateral compartments will induce displacement of the tibial component relative to the bone. The intention was to apply the maximal load of 20 N m. If the patient indicated that the load was too painful and/or uncomfortable, the load was reduced. The level of the load was registered.

##### Software: Quantifying and visualizing implant displacement

**Figure 2:**
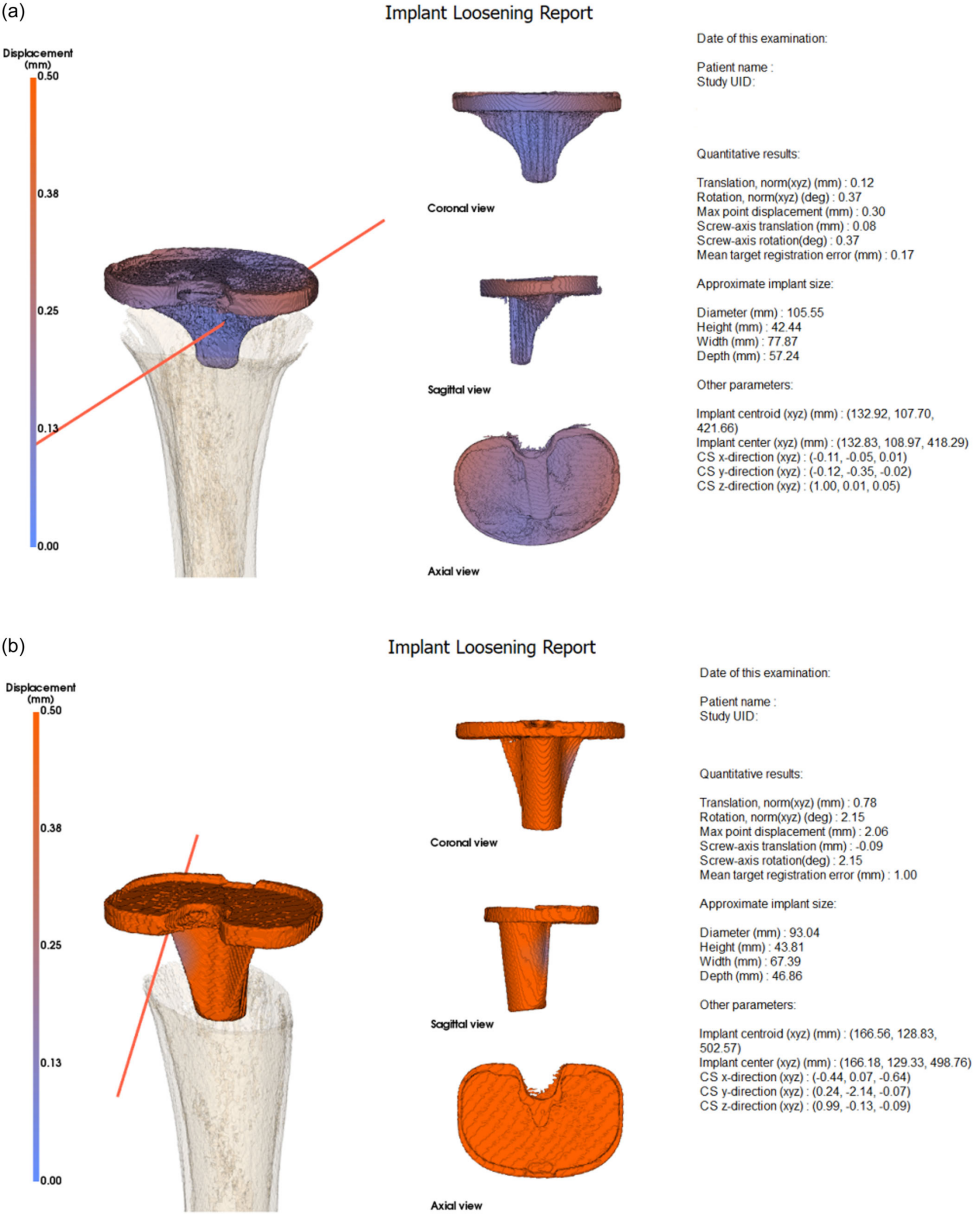
(a) Example of implant loosening report of a fixed tibial component. (b) Example of implant loosening report of a loose tibial component. deg, degrees; mm, millimetre.

Custom image analysis software was developed to quantify and visualize implant displacement. The methods incorporated in the software were based on a protocol by Dobbe et al. and were validated in a recently published cadaveric pilot study by Kievit and Buijs et al. [8, 14]. In short, the tibial implant and the tibial cortex were segmented by threshold‐connected region growing from the valgus image, resulting in a polygon mesh model of the implant and tibia used for visualization purposes. Image registrations from the tibial and implant segmentations to the varus 3D image were subsequently used to find the positions of the implant and tibia in the varus image. This enabled quantification of the relative displacement of the implant with respect to the tibia and expressing this displacement in terms of the following clinically relevant parameters: (1) rotation about the screw‐axis [19], (2) the average point displacement of all points in the implant mesh (mean Target Registration Error, mTRE [9]) and (3) the maximum point displacement observed across the implant model (maximum total point motion, MTPM) [14]. These parameters are shown in a report together with a visual representation of the implant and tibia. In addition, local point displacements of the implant model are visually represented by a heat map, showing colour variations indicating the magnitude of the local displacement (Figure 2a,b).

#### Reference test

Symptomatic patients underwent rTKA at their respective local hospitals. The orthopaedic surgeon performing the revision surgery was requested to assess the implant during the procedure. Based on the intraoperative observation, the surgeon classified the component as either ‘loose’ or ‘fixed’. This classification served as the reference for the study. The orthopaedic surgeons performing rTKA were blinded for the results of the index test.

#### Analysis

Diagnostic accuracy was evaluated first for all three selected displacement parameters separately (univariate) and then combined (multivariate). Based on the data from the symptomatic patients, optimal thresholds for individual displacement parameters were determined using univariate receiver operating characteristic (ROC) curves, with results providing sensitivity, specificity, positive predictive value (PPV) and negative predictive value (NPV) for each parameter.

Additionally, a multivariate logistic regression model was developed to determine the optimal threshold when combining the three displacement parameters. A multivariate ROC curve was plotted. The optimal threshold (closest to the upper left corner) derived from this multivariate ROC curve was computed and used to categorize predicted outcomes (loose or fixed). Subsequent accuracy metrics were then calculated.

To evaluate robustness, the model was checked for multicollinearity among the three predictors (rotation about the screw axis, mTRE and MTPM) using variance inflation factors (VIFs). A VIF greater than 10 was considered indicative of present multicollinearity that could affect the stability and interpretation of the model coefficients [15].

### Statistics

Inter‐ and intraclass correlation coefficients (ICCs) were calculated for rotation about the screw‐axis, mTRE and MTPM to assess interrater reliability, using a two‐way mixed‐effects (ICC3k) model for assessment of interrater reliability and a two‐way random‐effects model (ICC2k) for intra‐rater reliability. Moderate, good and excellent reliability were indicated by values of 0.5 to 0.75, 0.75 to 0.90 and >0.90, respectively [16].

Categorical variables were analysed using frequencies, proportions and Fisher's exact test. Parametric data were evaluated using mean, standard deviation (SD) and Student's *t* test. Non‐parametric data were assessed with median, interquartile range (IQR) and the Mann−Whitney *U* test.

The correlation between displacement parameters and KOOS (Knee Injury and Osteoarthritis Outcome Score) was analysed using the Spearman correlation coefficient (RS) [1].

Based on preliminary results, a logistic regression power analysis was performed (power: 0.85, odds ratio: 4.0, alpha 0.05 and beta 0.15), resulting in a required sample size of 37 symptomatic knees.

Data were analysed with R for Windows version 4.2.3 (R Foundation for Statistical Computing), using the ‘car’, ‘irr’, ‘psych’ and ‘pROC’ packages. A two‐sided *p* value < 0.05 was considered statistically significant.

## RESULTS

### Patient inclusion

From the Amsterdam UMC and seven affiliated hospitals, 36 patients, including 37 knees suspected of aseptic loosening, were referred to the Amsterdam UMC for induced displacement measurements. Of whom, two patients (three knees) did not receive a revision. Therefore, 34 knees of 34 patients were included for analysis in the symptomatic group (Table 1).

**Table 1:** Table of baseline characteristics.

	Symptomatic knees (*n* = 34)	Asymptomatic knees (*n* = 38)	*p* Value
Age (mean, SD)	67.9 (8.1)	68.8 (8.7)	0.29
Gender (*n*, %)			0.85
Male	18 (52.9)	22 (57.9)	
Female	16 (47.1)	16 (42.1)	
Time interval between scan and surgery (months [IQR])	0.9 (0.2–3.5)		
Type of prosthesis (*n*, %)			0.25
Regular	26 (76.5)	37 (97.4)	
Revision—short stem	2 (5.9)	0 (0.0)	
Revision—long stem	6 (17.6)	1 (2.6)	
KOOS subscores (median [IQR])			
KOOS‐Pain	33 (25–55.2)	97 (86.8–99.2)	<0.001
KOOS‐Other Symptoms	50 (36–64)	89 (82–95.2)	<0.001
KOOS‐ADL	41 (31.2–48)	91 (87–99)	<0.001
KOOS‐Sport/Rec	2.5 (0–15)	67 (45–85)	<0.001
KOOS‐QoL	18 (6.5–33)	81 (69–92.5)	<0.001
Magnitude of displacement (median [IQR])			
MTPM (mm)	0.86 (0.69–1.32)	0.64 (0.49–0.82)	0.001
mTRE (mm)	0.53 (0.14–0.82)	0.40 (0.29–0.50)	0.002
Rotation about screw‐axis (deg)	0.52 (0.51–1.28)	0.52 (0.40–0.67)	0.02

*Note*: Symptomatic knees are the groups “Symptomatic Loose” and Symptomatic Fixed” grouped together.

Abbreviations: ADL, function in daily living; deg, degrees; IQR, interquartile range; KOOS, Knee injury and Osteoarthritis Outcome Score; mm, millimetre; MTPM, maximum total point motion; mTRE, mean Target Registration Error; QoL, knee‐related quality of life; Sport/Rec, function in sport/recreation.

[Correction added on 17 July 2024, after first online publication: In Table 1, under the ‘Symptomatic knees’ column, the numbers in rows “Revision—short stem” and “Revision—long stem” have been corrected in this version.]

**Figure 3:**
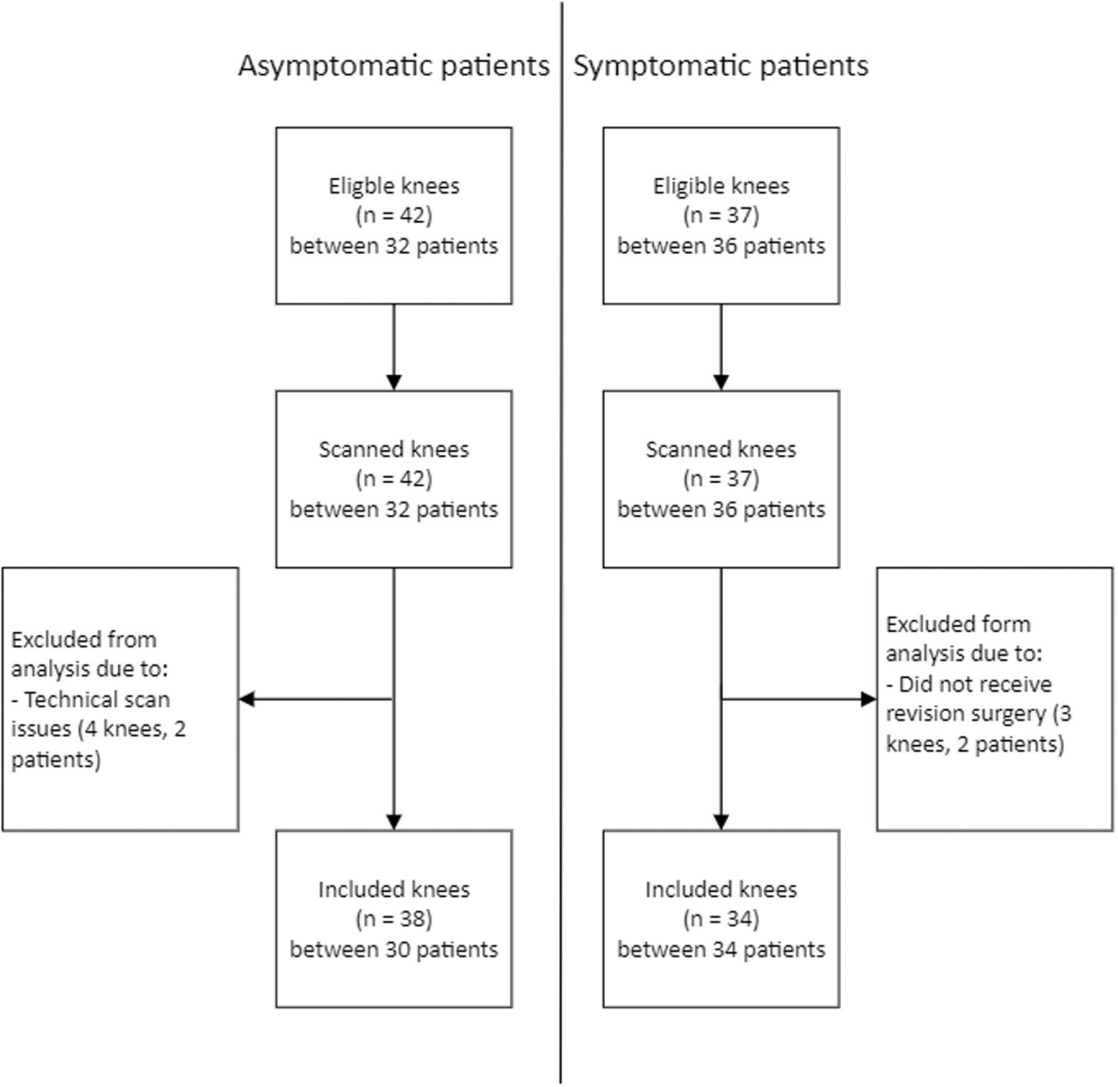
Flowchart of patient selection and inclusion.

In the asymptomatic group, 42 knees of 32 patients were scanned and evaluated. Four knees of two patients were excluded from the analysis because of technical issues (e.g., movement of the leg during scanning) (Figure 3, Table 1).

### Safety

Median NRS for pain and/or discomfort during loading was 6 (IQR: 3.75–7.00) and 2 (IQR: 1.00–3.00) and for symptomatic and asymptomatic patients, respectively. The median NRS for pain and discomfort after loading was 0 (IQR: 0.00–1.00) in asymptomatic patients and 3 (IQR: 0.75–5.25) in symptomatic patients. No adverse events were reported by the enroled patients.

### Feasibility

Ease of device positioning, strap attachment and application of moment were rated as easy in the majority of cases, with some difficulty noted in patients with high leg circumference. The mean moment applied in valgus was 18.9 Nm (SD: 2.92) and 19.0 N m (SD: 2.60) in varus. The target moment of 20 N m was reached in 62 (86.1%) cases in the valgus direction and 59 (80.6%) cases in the varus direction. Pain due to impingement of the straps into the skin was cited as the reason for not achieving the target moment in most cases. Median time spent in the CT room was 9 min (IQR: 8.00–11.00).

### Reliability

Good to excellent interrater correlation coefficients were found for rotation about the screw axis, mTRE and MTPM with medians 0.98 (IQR: 0.97–0.99), 0.89 (IQR: 0.84–0.93) and 0.93 (IQR: 0.90–0.96) respectively. Intra‐rater correlation coefficients were excellent (ICC: 0.91 [IQR: 0.64–0.98]) for rotation about the screw axis, excellent (ICC: 0.96 [IQR: 0.84–0.99]) for mTRE and moderate (ICC: 0.74 [IQR 0.02–0.94]) for MTPM.

### KOOS scores and magnitude of displacement

**Figure 4:**
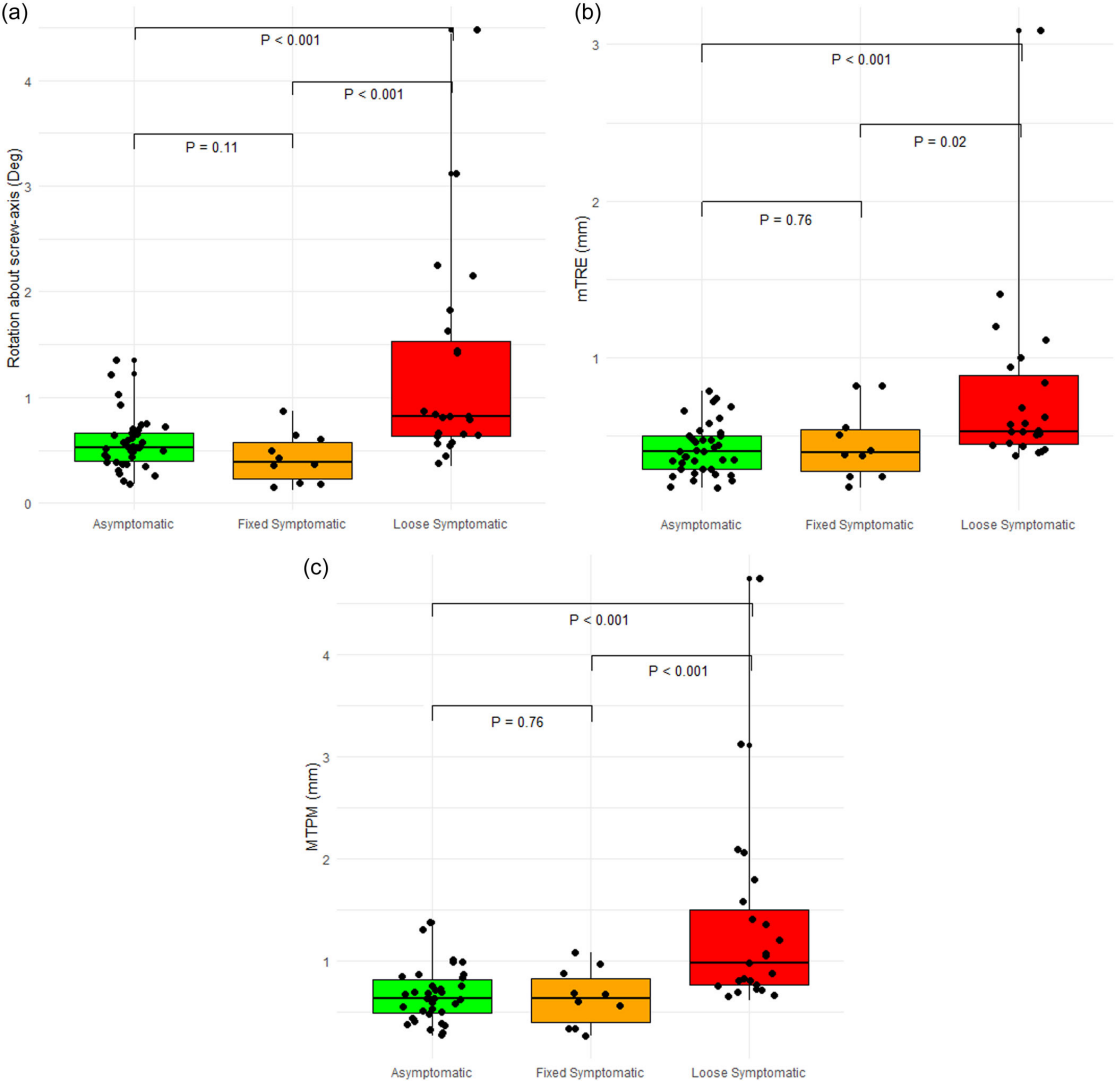
(a) Box‐ and scatterplot for rotation about screw‐axis in degrees (deg) per group. Median rotation about the screw axis was 0.52 (IQR: 0.40–0.67), 0.39 (IQR: 0.23–0.58) and 0.82 (IQR: 0.64–1.54) degrees for asymptomatic, symptomatic fixed and symptomatic loose knees, respectively. (b) Box‐ and scatterplot for mean Target Registration Error (mTRE) in millimetre (mm) per group. Median mTRE was 0.40 (IQR: 0.29–0.50), 0.40 (IQR: 0.28–0.54) and 0.53 (IQR: 0.45–0.89) mm for asymptomatic, symptomatic fixed and symptomatic loose knees, respectively. (c) Box‐ and scatterplot for maximum total point motion (MTPM) in mm per group. Median MTPM was 0.63 (IQR: 0.49–0.82), 0.64 (IQR: 0.40–0.83) and 0.98 (IQR: 0.77–1.51) mm for asymptomatic, symptomatic fixed and symptomatic loose knees, respectively. IQR, interquartile range.

Analysis of Spearman correlations between rotation about the screw‐axis, mTRE, MTPM and KOOS subscores resulted in almost all statistically significant negative correlations between the parameters and all KOOS subscores (Table 2). The magnitude of displacement is shown in Table 1 and Figure 4.

**Table 2:** Spearman correlations between movement parameters and KOOS subscores for all included patients.

	KOOS‐Pain	KOOS‐Other Symptoms	KOOS‐ADL	KOOS‐Sport/Rec	KOOS‐QoL
MTPM (mm)	−0.36 (*p* = 0.0023)	−0.29 (*p* = 0.013)	−0.36 (*p* = 0.0022)	−0.26 (*p* = 0.029)	−0.34 (*p* = 0.0041)
mTRE (mm)	−0.35 (*p* = 0.0031)	−0.30 (*p* = 0.012)	−0.34 (*p* = 0.0033)	−0.23 (*p* = 0.059)*	−0.30 (*p* = 0.012)
Rotation about screw‐axis (deg)	−0.28 (*p* = 0.018)	−0.23 (*p* = 0.053)*	−0.29 (*p* = 0.013)	−0.26 (*p* = 0.03)	−0.32 (*p* = 0.0061)

Abbreviations: ADL, function in daily living; KOOS, Knee injury and Osteoarthritis Outcome Score; MTPM, maximum total point motion; mTRE, mean Target Registration Error; QoL, knee‐related quality of life; Sport/Rec, function in sport/recreation.

* denotes not statistically significant.

### Diagnostic accuracy

**Figure 5:**
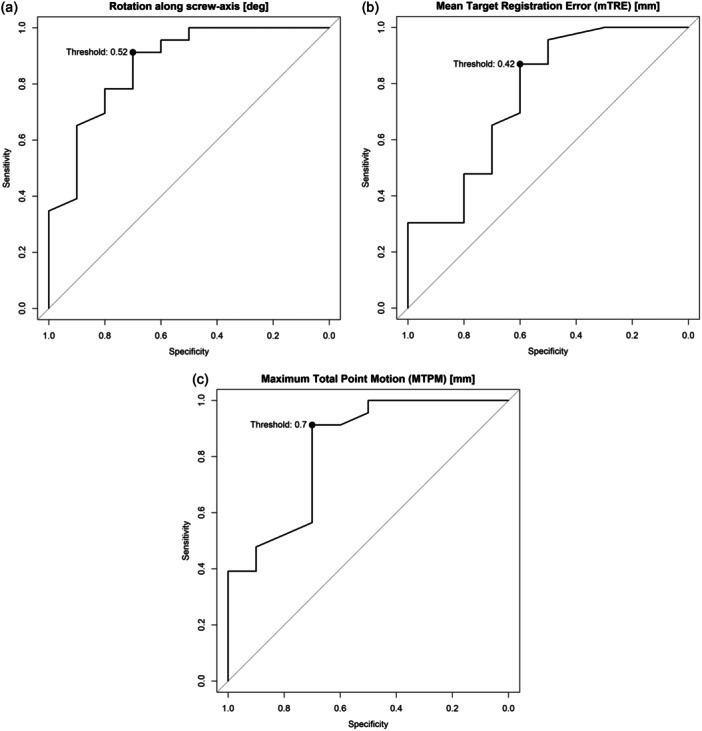
(a–c) ROC curves for rotation about the screw axis in degrees, mean Target Registration Error (mTRE) in mm, and maximum total point motion (MTPM) in mm, with optimal thresholds highlighted. ROC, receiver operating characteristic.

Based on separate ROC curves, optimal thresholds to differentiate between a loose and fixed tibial component were 0.53 degrees of rotation about the screw axis, a mTRE of 0.42 mm and a MTPM of 0.70 mm, respectively. Consequent diagnostic accuracy metrics and ROC curves are displayed in Figure 5 and Table 3.

**Table 3:** Results of uni‐ and multivariate accuracy metrics.

	Optimal Threshold	Sensitivity (95% CI)	Specificity (95% CI)	PPV (95% CI)	NPV (95% CI)
Rotation about the screw axis	0.53 deg	0.91 (0.52–0.91)	0.70 (0.30–0.70)	0.88 (0.68–0.97)	0.78 (0.40–0.97)
mTRE	0.42 mm	0.87 (0.35–0.87)	0.60 (0.20–0.60)	0.83 (0.63–0.95)	0.67 (0.30–0.93)
MTPM	0.70 mm	0.91 (0.35–0.87)	0.70 (0.30–0.70)	0.88 (0.68–0.97)	0.78 (0.40–0.97)
Multivariate regression model	0.57 (probability)	0.91 (0.72–0.97)	0.72 (0.43–0.90)	0.87 (0.68–0.95)	0.80 (0.49–0.95)

Abbreviations: CI, confidence interval; deg; degrees; mm; millimetre; MTPM, Maximum Total Point Motion, mTRE, mean Target Registration Error.

**Figure 6:**
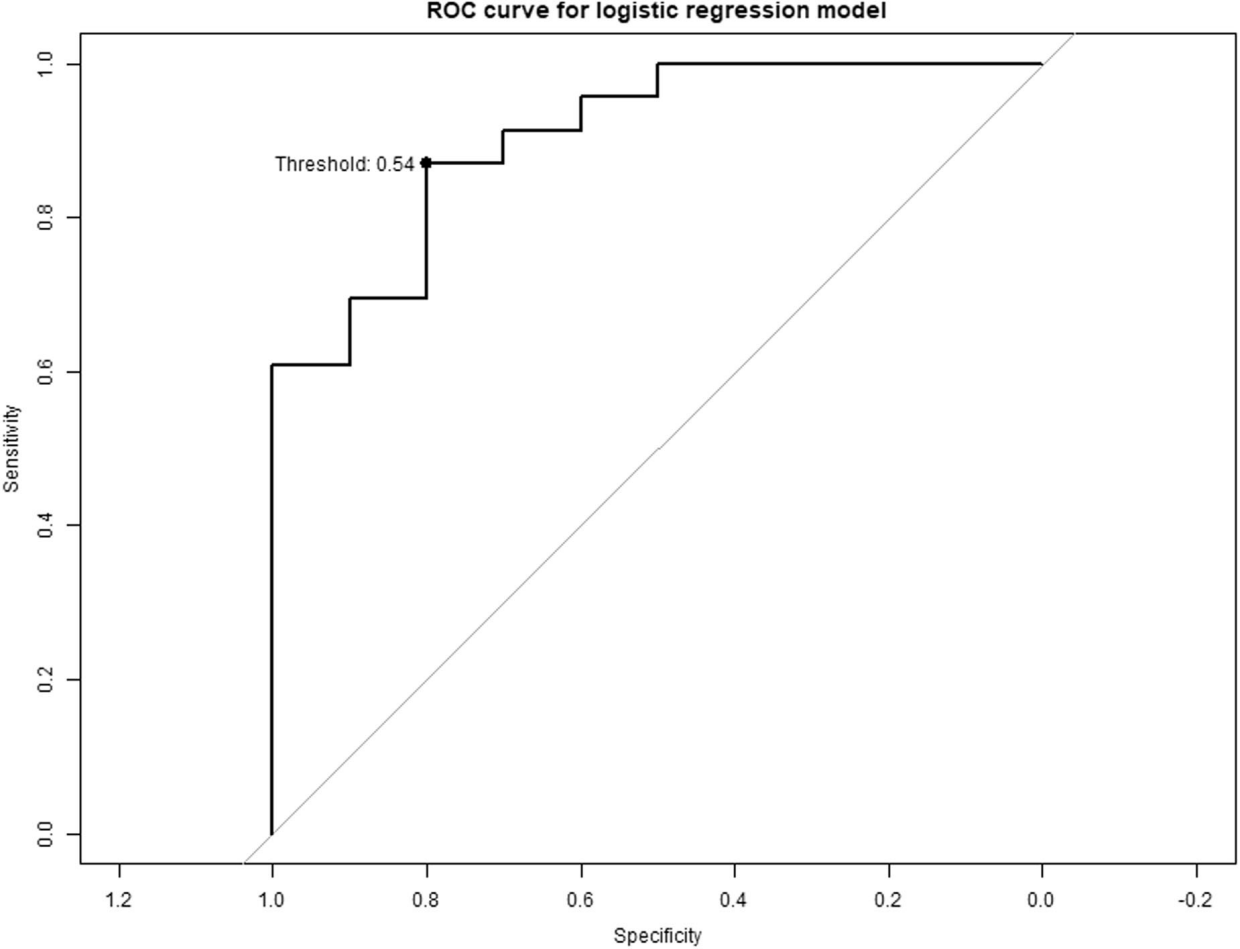
ROC curve of a multivariate logistic regression model with optimal probability threshold highlighted. ROC, receiver operating characteristic.

Combining all three parameters in a multivariate logistic regression model resulted in a VIF of 2.03 for rotation about the screw‐axis, 5.60 for mTRE and 7.79 for MTPM (Figure 6, Table 3). The multivariate ROC analysis yielded an AUC of 0.90 indicating excellent discriminatory ability. An optimal probability threshold of 0.54 was determined via the ROC analysis, maximizing the sensitivity and specificity trade‐off (Figure 6). At this threshold, the model exhibited a sensitivity of 0.91 (95% CI: 0.72–0.97), specificity of 0.72 (95% CI: 0.43–0.90), PPV of 0.87 (95% CI: 0.68–0.95) and NPV of 0.80 (95% CI: 0.49–0.95) (Table 3).

## DISCUSSION

The most important finding of this study is that the proposed non‐invasive modality is safe, feasible and reliable with a diagnostic accuracy that is better or at least as good as current diagnostic imaging modalities, such as CT, MRI and nuclear scans.

The induced displacement method demonstrated favourable safety, with tolerable pain and discomfort levels and no adverse events, highlighting its suitability for clinical use. It was found to be practical and easy for medical staff to use, even with the challenge of large leg circumferences in some patients. The method demonstrated high diagnostic accuracy, with 91% sensitivity and 72% specificity, underscoring its potential as a non‐invasive alternative to current modalities.

The outcomes of the asymptomatic patients were not statistically significantly different from the patients that were evaluated as fixed at the time of revision surgery. Referring to the KOOS outcomes, the asymptomatic patients did not have complaints, but it cannot be ruled out that these patients had a loose tibial TKA component. After all, 10 out of 38 asymptomatic knees would have been diagnosed as loose if applying the thresholds for rotation about the screw‐axis, mTRE and MTPM. Nevertheless, it is not yet established that a loose TKA may go undetected because of the absence of symptoms and additionally, the reported thresholds should be externally validated before clinical use.

Testing this technique against greater sets of databases enabling separate analysis for different types of implants could potentially increase and individualize the diagnostic accuracy of this method. It would be beneficial for future research to include the loosening of the femoral component and long‐term follow‐up of patients diagnosed with stable implants to confirm the absence of progressive loosening and to ensure that the method not only has diagnostic accuracy but also predictive validity. As an added value, TKA component migration over time can be measured and might serve as a less invasive alternative for model and/or marker‐based radiostereometric analysis.

Despite the positive findings, this study is not without limitations. First and foremost, the sample size needs to be expanded in future research to improve the robustness of the calculated thresholds. The current study evaluated the diagnostic accuracy in a diverse patient group with prostheses of different brands, materials and sizes and reported promising accuracy results. The current study did not differentiate between different TKA designs or types of fixations. To provide more detailed information on the extent of loosening for these different types, future studies should include a detailed evaluation of various implant designs, alignment and fixation techniques. Second, our current study involved 3D image analysis. During this process, we segmented and registered the whole tibia. The recent cadaveric study by Ter Wee et al. indicates that because of tibial deformation under loading, tibial component displacement is overestimated if the entire tibia is segmented [22]. Third, the comparison to the intraoperative ‘gold standard’ does not fully address the potential for surgeon bias or variation in intraoperative assessment of the fixation status of the tibial component. Finally, while patients with either tibial or femur component loosening report the same complaints (e.g., weight‐bearing pain), this study only addresses potential loosening of the tibial component (20.4% of the reasons for revision), yet neglects possible loosening of the femoral component (8.8% of reasons for rTKA) [10, 11].

## CONCLUSION

This non‐invasive method, which measures the induced displacement of the tibial TKA component has the potential as a safe, feasible, reliable and accurate diagnostic tool to aid the diagnosis of aseptic TKA loosening.

## AUTHOR CONTRIBUTIONS

George S. Buijs co‐designed and directed the project, performed patient inclusion, data acquisition and analysis and wrote the article. Arthur J. Kievit co‐designed and co‐directed the project, performed patient inclusion, data acquisition, supervised the analysis and cowrote the article. Maaike A. Ter Wee acted as an interrater in the analysis. Caroline Magg acted as an interrater in the analysis. Johannes G. G. Dobbe supervised the software analysis. Geert J. Streekstra supervised the software analysis and helped with data acquisition. Matthias U. Schafroth helped with patient inclusion, data acquisition and supervised the whole project. Leendert Blankevoort performed patient inclusion, data acquisition and directed the project. All authors discussed the results and contributed to the final manuscript.

## CONFLICT OF INTEREST STATEMENT

Authors Leendert Blankevoort, Arthur Kievit and Matthias Schafroth are listed as inventors on a patent for a loading device that can be used to quantify and visualize implant displacement and own minority stock in an Amsterdam University Medical Center spinoff company called AtMoves B.V.

## ETHICS STATEMENT

This study (NL49757.018.14) was approved by the institutional review board of the Amsterdam University Medical Center (Amsterdam UMC). Informed consent was required before participation. This prospective trial was registered at the trial registry of the Central Committee for Research with Human Subjects (CCMO; NL49757.018.14).

## Data Availability

The data that support the findings of this study are available on request from the corresponding author.
